# The Effect of surface topographical changes of two different surface treatments rotary instrument

**DOI:** 10.4317/jced.54472

**Published:** 2018-01-01

**Authors:** Nashwan-Ahmed Qaed, Bilal-Diab Mourshed, Hashem-Motahir Al-Shamiri, Nader Alaizari, Saleh-Sulaiman Alhamdah

**Affiliations:** 1 BDS, MSc, Lecturer, Department of restorative Dentistry, Al-Farabi Colleges, Riyadh, Saudi Arabia; 2 BDS, MSc, Lecturer, Department of Prosthetic Dental Sciences, Al-Farabi College for Dentistry and Nursing, Riyadh, Saudi Arabia; Department of Prosthodontics, Propaedeutics and Dental Materials, School of Dentistry, Christian-Albrechts University at Kiel, Kiel, Germany; 3 BDS, MSc, Lecturer- Department of Oral and Maxillofacial Surgery, Al-Farabi Colleges, Riyadh, Saudi Arabia; 4 BDS, MSc, Lecturer, Department of Oral Medicine and Diagnostic Sciences, AlFarabi Colleges, Riyadh, Saudi Arabia; 5 BDS, Al-Farabi Dental College, Riyadh, Saudi Arabia

## Abstract

**Background:**

One of the major innovations in endodontics has been the introduction of nickel-titanium (NiTi) alloy. This study evaluated the surface topographical changes of two different surface treatments rotary instrument after instrumentation and sterilization.

**Material and Methods:**

240 Extracted teeth were included in this study. 90 new AlphaKite and Revo-S NiTi rotary instruments were selected and divided into two groups (Group A 45 AlphaKite and group B 45 Revo-S). Each group were divided into three subgroups: (A1, B1) n=5 files were used as a control, (A2,B2) n=20 files were used to prepare three root canals using endodontic rotary motor then sterilized by autoclave for one cycle under 121°C at 15 psi for 30 minutes and (A3,B3) n=20 files were used to prepare nine root canals using the same rotary system then sterilized by autoclave for three cycles under 121°C at 15 psi for 30 minutes. Files were examined under scanning electron microscopy.

**Results:**

On examining the AlphaKite, A1 revealed gross machining grooves on their surface with no pits, A2 showed disruption of cutting and A3 showed microcracks and deepening of the machining grooves. B1 showed a smoother surface with few machining grooves, B2 showed dulling and blunting of the cutting edges was predominant and B3 files showed plastic deformation in the form of unwinding of the flutes.

**Conclusions:**

The defects were less distributed along the electropolished Revo-S files than the physical vapor deposition AlphaKite.

** Key words:**Endodontic instruments, electropolished, rotary, sterilization.

## Introduction

One of the major innovations in endodontics has been the introduction of nickel-titanium (NiTi) alloy to manufacture root canal instruments with a reduction of working time (1) and avoiding the disadvantages of traditional instruments and devices (2,3).

During the treatment of root canal, NiTi rotary instruments may fracture mostly due to exceed the maximum number of usage recommended by the manufacturer (4), thereby it may jeopardize the outcome of the root canal treatment. The fracture of NiTi rotary instruments occurs due to two different mechanisms which are torsional fracture and flexural fatigue (5) because of that, some authors recommend considering the file as a disposable instrument which should be used for only once to overcome its inherent weakness (4). But owing to the high cost of rotary instruments, these NiTi files are frequently reused after sterilization by autoclaving which minimize the cost and risk of cross-infection during endodontic treatment.

Thus a question should be answered whether the surface topography of rotary nickel titanium files will be affected by instrumentation and different cycling of sterilization or not.

## Material and Methods

-Selection and Preparation of Teeth

240 Extracted mandibular first molars with only two curved mesial roots ranging from 10 to 30 degrees measured according to Schneider’s technique were included in this study (6). The lengths of the mesial canals ranging from 14-16 millimeter were selected. The teeth were cleaned and stored in sterile saline at room temperature till the time of use. The distal root of each tooth was sectioned and removed. After gaining access, each mesial root canal were checked for apical patency by inserting a size #10 stainless steel K file (Mani Matsutani Seisakusho Co. Takanezaw Machi Tochigi Ken Japan). Working length was established by subtracting one millimeter from that measurement. Canals were scouted with #15 K file before using the engine driven rotary nickel titanium instruments.

The crowns of all teeth were decapitated at the level of 3mm above the cementoenamel junction utilizing a thin safe sided diamond disc mounted on a conventional speed contra angle hand piece (J Morita Mfg. Corp. Japan) under water coolant.

120 teeth with 240 mesial root canals were prepared for each Revo-S (Micromega, France) and AlphaKite (Gebr. Brasseler, Germany) NiTi rotary instruments.

-Instruments and preparation techniques 

90 new AlphaKite and Revo-S NiTi rotary instruments with 0.04 taper were selected and divided into two groups (Group A 45 AlphaKite and group B 45 Revo-S). Each group of instruments were then divided into three subgroups: (A1, B1) subgroup files (n=5) were used as a control, (A2,B2) subgroup files (n=20) were used to prepare three root canals using dentaport endodontic rotary motor then sterilized by autoclave for one cycle under 121°C at 15 psi for 30 minutes and (A3,B3) subgroup files (n=20) were used to prepare nine root canals using the same rotary system then sterilized by autoclave for three cycles under 121°C at 15 psi for 30 minutes.

-Qualitative Evaluation

All files were evaluated qualitatively for surface changes at the tip, shaft and the edges of the flutes using the environmental scanning electron microscopy (ESEM) at magnification 200X-1000X by two observers.

## Results

**Figure 1:**
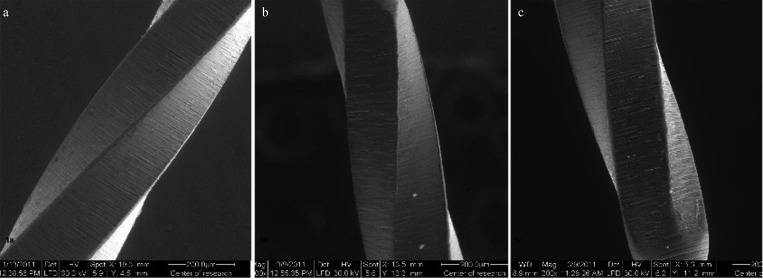
(a): Scanning electron micrograph of unused AlphaKite file (subgroup A1) with marked machining grooves (X300-Magnification) (b): Scanning electron micrograph of AlphaKite file after (subgroup A2 ) presenting disruption and blunting of cutting edges and deepening of the machining groove (X300 magnification) (c): Scanning electron micrograph of AlphaKite file ( subgroup A3) showing microcracks (white arrow) and deepening of the the machining grooves with metal stripping along the cutting edge (black arrow) (X300 magnification).

On examining the AlphaKite files subgroup A1, the unused group, under ESEM, revealed gross machining grooves on their surface with no pits, strips or disruption (Fig. 1a).

After three uses and one autoclave cycle, subgroup A2, scanning electron micrographs showed disruption of cutting edges that were also blunted and scraped with deepening of the machining grooves (Fig. 1b).

**Figure 2:**
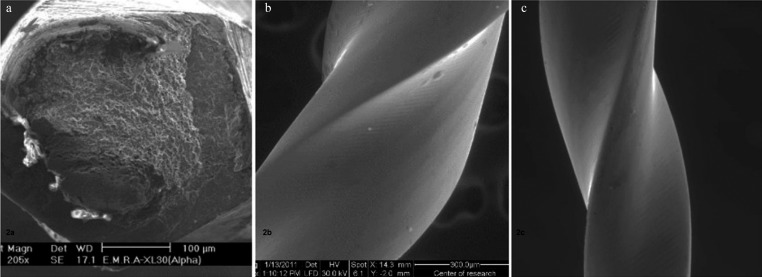
(a): Scanning electron micrograph of AlphaKite file (subgroup A3) showing macroscopic plastic deformation at the fracture site (black arrow). Signs of ductile fracture, and irregular dimples that resembles a fibrous structure (white arrow) (X205 magnification) (b): Scanning electron micrograph of unused Revo-S file (subgroup B1) with smooth surface and slight machining groove (X300 Magnification) (c): Scanning electron micrograph of Revo-S file (subgroup B2) showing dulling and scrapping of the cutting edges (X300 magnification).

After nine uses and three sterilization cycles in autoclave, subgroup A3, the scanning electron micrographs showed microcracks and deepening of the machining grooves with metal stripping along the cutting edge (Fig. 1c). In this group also one of the instruments was fractured. The fractured surface under magnification (X 205) showed the classical signs of ductile fracture; plastic deformation with areas showing irregular dimples and concentric abrasion marks at the periphery with a fibrous appearance in the center (Fig. 2a).

By examining Revo-S files, the scanning electron micrographs of unused files, subgroup B1, showed a smoother surface with few machining grooves (Fig. 2b).

On examining Revo-S files after three uses and one sterilization cycle, subgroup B2 , dulling and blunting of the cutting edges was predominant (Fig. 2c).

**Figure 3:**
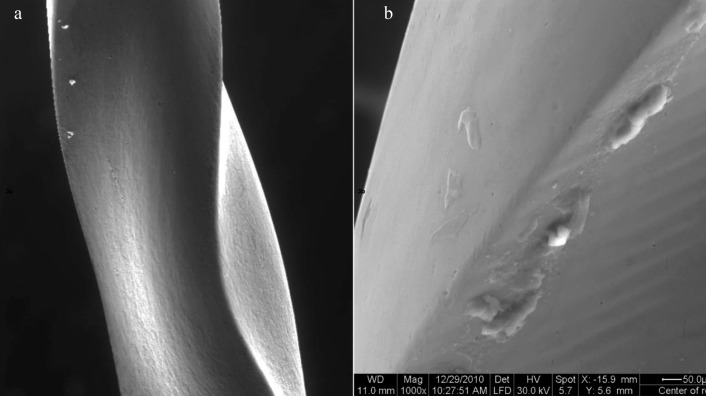
(a): Scanning electron micrograph of Revo-S file (subgroup B3) showing unwinding (x200 magnification) (b): Scanning electron micrograph of Revo-S file (subgroup B3) showing metal flakes on the file surface (X1000 magnification).

After nine uses and three sterilization cycles, subgroup B3, files showed plastic deformation in the form of unwinding of the flutes, disruption in the surface with metal flakes (Fig. 3a,b).

## Discussion

This study aimed to evaluate the cumulative effect of instrumentation, irrigation and sterilization on the surface topography of two brands of endodontic rotary NiTi file systems.

In this study the rotary nickel titanium instruments were used to prepare three canals (which is considered to be a single molar), and nine canals (which is considered to be three molars) of mesial roots of first mandibular molar (7). The mesial canals of mandibular molars are frequently small and curved in three dimensions (8).

Cleaning, disinfection and sterilization procedure minimize and prevent cross infection, the files in this study were cleaned by ultrasonic bath to remove any biological debris before sterilization by autoclave (9). The number of sterilization cycles corresponds to the number of use, thus one cycle of sterilization was done after three canals in subgroup A2 and B2. While in subgroup A3 and B3, files were sterilized in autoclave for three cycles presenting three clinical cases.

In our study the ESEM of unused AlphaKite files showed the lowest distribution of defects among other groups of the same brand. These defects were represented mainly as machining grooves. This result was similar to other studies which found that all the new files presented surface imperfections including AlphaKite (10,11).

Present manufacturing technology for rotary nickel titanium instruments seems to be incapable of avoiding the creation of surface flaws and rollover, which appears during machining of the highly flexible NiTi (7). It is noticeable that such topographic irregularities may have a considerable impact on mechanical properties of endodontic files (12) and make them more powerless to fracture (13).

Manufacturing defects in the form of minute crossing fissures on the surface of the unused files could be due to that NiTi instruments are machined directly from nitinol wire where their machining is generally difficult, as burs used during the manufacturing process become blunt and dull rapidly causing surface imperfections which may explain the presence of surface defects on unused NiTi rotary files (14).

Meanwhile scanning electron micrographs of the unused Revo-S files revealed smooth surface along the file length with no evidence of machining striations. According to the manufacturer, the Revo-S files is shown with electrochemical polishing that it receives, giving it a smooth characteristic with a low incidence of defects. The study of Arantes *et al.* (3) confirmed that the presence of defects was clear with all type of instrument except with files which was polished electochimically. However, it was also noted that the instruments that were not polished electrochemically presented a greater number of manufacturing defects which leading to fracture of the instrument during work (15,16) and those that were polished electrochemically had a better performance, which is associated with fewer irregularities that focus the instruments’ points of stress.

In addition, electropolishing appears to have a beneficial impact by enhancing cyclic fatigue and peak torque values for NiTi instruments to produce a surface with fewer structural defects that could enhance fracture resistance (17,18).

Both AlphaKite and Rivo S NiTi files showed changes their cutting edges after 3 uses and one autoclave cycle. As shown in Fig. 1b AlphaKite files under the scanning electron micrographs showed disruption of cutting edges that were blunted and scraped with deepening of the machining grooves while Revo-S files, the dulling and blunting of the cutting edges was predominant (Fig. 2c).

The incidence of these observations increased with increase in the number of usage times, these results were in agreement with Arantes *et al.* (3) which indicated that all instruments showed changes in their cutting blades after five uses but were contradicted by Rapisarda *et al.* 2001 (19) whose conclusion was ionic implantation increases the life span of the instrument, maintaining its precision and shape even after repeated use inside endodontic simulators, however it should be noted that Rapisarda *et al.* used ProFile instruments (#25 taper 0.04 ) where the surface was treated with a different method than the cathodic arc evaporation technique and they applied the study on resin blocks in place of natural teeth without using sodium hypochlorite or autoclave for sterilization.

The AlphaKite files after nine uses demonstrated severe wear, rolling, pitting and irregularities along the cutting edges of the flutes with widening of the machining grooves in addition to the presence of cracks and microfractures along the cutting edges of the file, despite all of these defects, only one AlphaKite rotary nickel titanium file showed complete fracture, indicating that the number of usage times was not too high and was within the range recommended by the manufacturer.

Instrument separation occurs mainly due to two factors namely the flexural cyclic fatigue and the torsional failure. When an instrument tip or another part of the instrument is locked in the canal and the shank continues to rotate thus the elastic limit of the metal is exceeded by the handpiece , and torsional fracture occurs (20). However fracture caused by flexural cyclic fatigue that occurs when the instrument does not bind in the canal, but it rotates freely in a curvature, generating tension/compression cycles at the point of maximum flexure until the fracture occurs (20,21). On examining the cross sectional area of the fractured file it was clear that the file presented fracture mainly due to dominant torsional stresses. The presence of microcracks, pitting and microfractures indicated that the used instruments had been worn. In fact the presence of these defects suggested an increased potential for failure with further use because the defects could act as local stress raisers and a potential origin for crack propagation (22,23).

In the study of Pirani *et al.* (10) four different NiTi files were tested in the curved of 45 and 60. AlphaKit showed a high flexural resistance after NTR files, however the study was subjected to artificial canals until fracture. Patino *et al.* 2005 (24) reported that instruments used more than eight times (in canals with mean curvatures of about 40 degrees) fractured more frequently than those used sparingly. This may raise the hypothesis of using nickel titanium rotary files as a disposable especially the small number Because it was hard to detect minor defects and fractures (4).

On the other hand, the surface changes of the Revo-S files after 9 uses were mainly in the form of plastic deformation represented by unwinding of the flutes and blunting or disruption of the cutting edges with increased pitting. This enhanced performance could be explained by the fact that the Revo-S has small triangular cross section with decreased metal core besides the effect of smoothening obtained by electropolishing thus reducing the stress applied on the surface of instruments (25). In addition, by removing the surface layer of the instrument by electropolishing this may potentially eliminate residual stresses which might explain the absence of any fractured Revo-S file (15).

## Conclusions

Multiple use of endodontic files results in different surface defects increased with increase in the number of usage times and surface treatment by electropolishing adopted by manufacturer to improve files performance was more efficient than physical vapor deposition.
